# Genome-Editing Technologies for Enhancing Plant Disease Resistance

**DOI:** 10.3389/fpls.2016.01813

**Published:** 2016-12-01

**Authors:** Giuseppe Andolfo, Paolo Iovieno, Luigi Frusciante, Maria R. Ercolano

**Affiliations:** Department of Agricultural Sciences, University of Naples ‘Federico II’Portici, Italy

**Keywords:** defense system, plant immunity, IAC, IMC, genome editing, effector, *R*-gene

## Abstract

One of the greatest challenges for agricultural science in the 21st century is to improve yield stability through the progressive development of superior cultivars. The increasing numbers of infectious plant diseases that are caused by plant-pathogens make it ever more necessary to develop new strategies for plant disease resistance breeding. Targeted genome engineering allows the introduction of precise modifications directly into a commercial variety, offering a viable alternative to traditional breeding methods. Genome editing is a powerful tool for modifying crucial players in the plant immunity system. In this work, we propose and discuss genome-editing strategies and targets for improving resistance to phytopathogens. First of all, we present the opportunities to rewrite the effector-target sequence for avoiding effector-target molecular interaction and also to modify effector-target promoters for increasing the expression of target genes involved in the resistance process. In addition, we describe potential approaches for obtaining synthetic *R*-genes through genome-editing technologies (GETs). Finally, we illustrate a genome editing flowchart to modify the pathogen recognition sites and engineer an *R*-gene that mounts resistance to some phylogenetically divergent pathogens. GETs potentially mark the beginning of a new era, in which synthetic biology affords a basis for obtaining a reinforced plant defense system. Nowadays it is conceivable that by modulating the function of the major plant immunity players, we will be able to improve crop performance for a sustainable agriculture.

## An Important Reason for Enhancing the Plant Immune System

The principal aim of sustainable intensification of agriculture is to increase food production while minimizing pressure on the environment. Phytopathogens limit crop yields and pose a threat to food sustainability worldwide. In the absence of genetic resistance, crop production relies heavily on chemical control of pathogens. Reducing the dependence of food production on chemical control is a key goal for avoiding negative environmental impacts caused by current practices (Tilman et al., 2002) and taking significant global climate change into account (IPCC, 2007). Plant domestication and breeding processes allow crops to be obtained with improved performance and tailored traits. The most renewable strategy to manage plant disease is to develop resistant plants, thereby obtaining environmental, economic, and social benefits. Therefore, the genes for resistance to pests and diseases can be rightfully considered essential resources to meet human food requirements (Mundt, 1994).

## Plant Immunity Components

Plants have developed a plethora of defense mechanisms underlying disease suppression to ward off damage caused by pathogens. The response of plants to pathogen attack relies on pathogen recognition at the cellular level, which then triggers complex signaling pathways (Jones and Dangl, 2006; Andolfo and Ercolano, 2015). When the plant perceives the signals of danger as pathogen-associated molecular patterns (PAMPs) or damage-associated molecular patterns (DAMPs), effectors, prompt the stereotypical defense program (Wise et al., 2007). The plant innate immune system is based on two distinct but interconnected components, namely the immunity activation component (IAC) and the immunity modulation component (IMC). The IAC is based on a large number of surveillance receptors: pattern-recognition receptors (PRRs) and Nibblers (NB-LRR receptors) that recognize the presence of pathogens and convey the message of invasion. The second component (IMC) is based on the phytohormones that play a fundamental role in regulating plant immune response (Shah, 2003; von Essen et al., 2010).

In an exemplified model, three distinct stages (1: interaction, 2: activation/modulation, and 3: effective resistance/immunity) can be identified in a generic plant-pathogen interaction (Andolfo and Ercolano, 2015). During the first stage, the conformation of virulence factor targets is modified and several alterations of primary plant metabolism are detected. In the second stage, modification of virulence factor targets induces the Nibblers/PRR-triggered signaling (NTS and PTS). Furthermore, a feedback regulation of primary metabolisms, mediated by the metabolic alterations, induced a hormone-tempered resistance (HTR). In the effective resistance/immunity stage, the NTS/PTS, and the HTR converge to confer a pathogen lifestyle-specific resistance (PSR).

Rapid adaptation to threats is orchestrated by a complex regulatory network of interconnected signaling pathways. The newly emerging picture indicates that complex crosstalk among different classes of hormones might modulate disease resistance, with outcomes dependent on pathogen lifestyles and the genetic background of the host (Andolfo and Ercolano, 2015). Plant defense systems have been extensively investigated in the last decades, but exactly how they recognize pathogens and how IAC and IMC are regulated remains unknown. During the plant immunity process several gene networks are established following signaling cascades, in which regulators must fine-tune their activity to cooperate with or antagonize other regulators. Although pathogens have evolved to hijack this highly interconnected network of regulators to promote their virulence (Grant and Jones, 2009), emerging evidence suggests that crosstalk between immunity regulators offers the potential to fine-tune plant defense responses. Knowledge of host receptors variation should be combined with complementary knowledge of PMAPs/Effectors variation in the pathogen in order to provide effective new resistance genes. The large number of genes that are thought to be involved in the resistance process complicates our understanding of the biological molecules and pathways involved. Naturally variable alleles in pathogen receptor genes and downstream components of the resistance process, such as mitogen-activated protein kinases, transcription factors, and proteases/lipases, have been shown to contribute to disease resistance (Druka et al., 2008; Chen et al., 2010; Moscou et al., 2011; Camañes et al., 2012; Corwin et al., 2016). Identification of such variants may help engineer long-lasting and broad-spectrum disease resistance in crops with both durable resistances to pathogens and increased yields (Dangl et al., 2013).

Following classical breeding methodologies, we can introgress the resistance traits, making use of natural genetic variation, through several rounds of genetic recombination. New alleles can be introduced by random mutagenesis, although this is usually followed by the time-consuming screening of large populations to identify mutants (Parry et al., 2009; Sikora et al., 2011). Genome-editing technologies (GETs) allow site-specific mutagenesis to be achieved, overcoming the limits imposed by previous methods. Indeed, plant disease resistance can be increased by targeting suitable actors of plant defense machinery. However, to extend GET applicability to the ever-increasing number of crops some bottlenecks should be solved. In this work we describe suitable methods with potential pitfalls and crucial targets for engineering plant immunity.

## Targeted Genome Engineering Techniques

Site-directed mutagenesis relies on the introduction of targeted DNA double-strand breaks (DSBs) by action of programmable nucleases. Small deletions, targeted insertions, and multiplex genome modifications can result both from non-homologous end joining (NHEJ) and homologous recombination (HR) cellular DNA repair mechanisms. Artificial zinc-finger nucleases (ZFNs; Kim et al., 1996) and transcription activator-like effector nucleases (TALENs; Christian et al., 2010) contain a DNA cleavage domain from the restriction enzyme Fok I fused to an engineered DNA-binding domain. The CRISPR (Clustered Regularly Interspaced Short Palindromic Repeats)/Cas9(CRISPR-associated protein-9 nuclease) is based on RNA-guided engineered nucleases. Such genome-editing technology holds great promise due to its simplicity, efficiency and versatility (Jinek et al., 2012). CRISPR/Cas9 cleavage coupled with homology-directed repair (HDR) has the potential to enable engineering of new alleles of endogenous genes or the sequential insertion of transgenes at the same locus (Feng et al., 2014). Moreover, the CRISPR/Cas9 system is advantageous over ZFNs and TALENs since it allows simultaneous editing at multiple sites across the genome (Cong et al., 2013).

The applicability of GETs in the field of plant biology was already demonstrated in the model species *Arabidopsis thaliana* (Christian et al., 2010; Osakabe et al., 2010; Cermak et al., 2011; Li et al., 2013) and *Nicotiana benthamiana* (Nekrasov et al., 2013; Gao et al., 2015) as well as in other crops including rice, sorghum, wheat, corn, soybean, tobacco, potato, petunia, sweet orange, liver worth, and poplar (Shan et al., 2014; Luo et al., 2016; Rani et al., 2016). Stable inheritance of homozygous mutations induced by GETs and segregation of the mutation in the off springs was reported in several species (Maeder et al., 2008; Christian et al., 2010; Zhang et al., 2010; Qi et al., 2013; Brooks et al., 2014; Fauser et al., 2014; Feng et al., 2014; Jia et al., 2014; Schiml et al., 2014; Zhang et al., 2014; Zhou et al., 2014; Forner et al., 2015). GETs have been shown to be excellent tools to engineer metabolic circuits for synthetic biology applications (Bortesi and Fischer, 2015) since gene expression could be modulated by an inactive nuclease fused with either transcriptional activation or repression domains (Gilbert et al., 2013; Maeder et al., 2013; Bortesi and Fischer, 2015). Indeed, the transcription of a *PDS* gene of *N. benthamiana* was modified by Piatek et al. (2014). With the availability of an increasing amount of genomic data, genome engineering is developing increasingly precise methodologies. There is no general strategy for obtaining successful modifications. *In silico* approaches can help to predict candidates for resistance (Sanseverino and Ercolano, 2012) and to select target site, minimizing the occurrence of off-targets (Peng et al., 2016; Rani et al., 2016). Identification of amino acid residues under selective pressure can also provide valuable support (Iovieno et al., 2015). In order to reduce off-target activity two separate sgRNA target sequences can be used to guide a Cas9 nickase variant to two adjacent positions in the genome. Indeed, the enzyme induces a single-strand break (SSB) in each of the two DNA strands, increasing cleavage specificity (Schiml and Puchta, 2016). Optimization of delivery methods, HDR incidence and enzyme activity could also increase editing efficiency and specificity (Peng et al., 2016). Finally, genetic transformation and plant regeneration are bottlenecks in plant editing of several species, in particular tree species and need to be improved to extend the use of GETs in important crops (Luo et al., 2016).

## Genome-Editing Applications for Plant Disease Resistance

The growing need for crop yield stability has prompted breeding research to design plants able to respond to pathogen attacks without fitness penalties. GETs have been employed to modify major players of plant immunity at different levels in several crops. Host susceptibility genes (S-genes) have been successfully manipulated to promote resistance to key pathogens. TALEN and CRISPR/Cas9 technologies were both used to target the mildew-resistance locus O (*MLO*) in wheat (Wang et al., 2014), generating plants resistant to powdery mildew disease. GETs were used to generate plants resistant to bacterial leaf blight, caused by *Xanthomonas oryzae* pv. *oryzae*, impairing down the transcriptional regulation of S-genes by the effector. Indeed, the plants stably edited in the *OsSWEET14* promoter were resistant to bacterial strains since the effector was unable to activate the transcription of its target (Li et al., 2012). The metabolic pathways that regulate hormonal balance can be modified to enhance the IMC component of plant immunity. This goal was achieved by using GETs to cause the down-regulation of ethylene-responsive factors (*ERF*). In particular, the ethylene pathway in rice was successfully modified to increase resistance to *Magnaporthe oryzae* (Liu et al., 2012), using CRISPR/Cas9 technology to target a mutation in OsERF922 (Wang et al., 2016). In addition, the utility of GETs to introduce resistance was also demonstrated by the deletion of a host factor not directly involved in IMC but strictly required for pathogen survival (Pyott et al., 2016).

Furthermore, a new player can be introduced into plant immunity by using GETs. Indeed, the portability of the CRISPR/Cas9 system was demonstrated for introducing a new source of resistance to the geminivirus, circular single-stranded DNA (ssDNA) viruses that replicate within the nuclei of plant cells, causing serious damage to many dicotyledonous crop plants such as beet severe curly top virus in *Arabidopsis* and *N. benthamiana* (Ji et al., 2015) and bean yellow dwarf and tomato yellow leaf curl virus in *N. benthamiana* (Ali et al., 2015; Baltes et al., 2015). When plant immunity can target the D/S DNA replicative form, using CRISPR/Cas9 in a similar way to its endogenous role in Archaea, it is possible to trigger mutations and interfere with the copy number of freely replicating viruses. CRISPR/Cas9 was also used to knock down the eIF(iso)4E gene encoding for a translation complex in cucumber (*Cucumis sativus*). The induced mutation in the host eIF(iso)4E, confers resistance to cucumber vein yellowing virus (CVYV), zucchini yellow mosaic virus (ZYMV), and papaya ringspot virus-type W (PRSV-W) (Chandrasekaran et al., 2016). A novel approach to developing therapies for infectious diseases is to block bacteria, without killing them. In *Phytophthora sojae*, the possibility of editing a pathogen gene (Avr4/6) involved in the immunity activation was efficiently proved (Fang and Tyler, 2016).

## Opportunity to Obtain a Synthetic *R*-Gene for Single or Multi-Resistance

Traditional resistance breeding is based on the introgression of resistance traits, such as NLR (nucleotide-binding, leucine-rich repeat) genes, from wild species into elite varieties (Ercolano et al., 2012; Andolfo et al., 2014). Genetic variation for disease resistance within a plant is most often explained by allelic variation in the receptor encoding genes. Unfortunately, *R*-gene-mediated resistance is based on recognition of a single elicitor and the frequency of resistance breakdown is typically high. Therefore, a continuous influx of novel resistance genes in breeding programs is required. Recent transgenic strategies also allow the efficient transfer of *R*-genes between plant species (Faino et al., 2010; Horvath et al., 2012; Narusaka et al., 2013). However, the deployment of novel resistance genes through both conventional breeding and transgenic approaches is hampered by the low occurrence of *R*-genes with the useful response specificities. Specific *R*-gene targets could be edited since one or few polymorphic amino acids in the coiled-coil (CC) and/or nucleotide-binding (NB) domain are known to be responsible for recognition specificity (Ashikawa, 2012). In addition, it was observed that a double aminoacidic mutation enhanced the ability of the *R*-protein to trigger cell death (Stirnweis et al., 2014).

**FIGURE 1:**
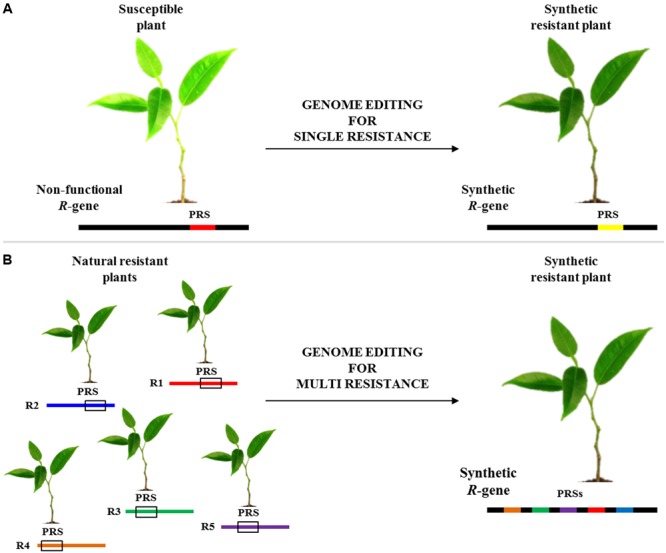
**Flowchart of disease resistance genes editing. (A)** Modification of a non-functional pathogen recognition site (PRS; red line) to obtain a synthetic functional *R*-gene (yellow line). **(B)** Another potential use of editing technology is the engineering of a novel synthetic *R*-gene able to mount resistance to several pathogens by combining PRS from different *R*-genes (R1-R5).

A recent work demonstrated that synthetic immune receptors (I2) can be engineered to confer resistance to phylogenetically divergent pathogens (Giannakopoulou et al., 2015). GETs could be very useful for designing and engineering *R*-genes with novel activities, where mutants identified in one gene could be transferred to homologs (**Figure 1A**). As shown in **Figure 1B**, genome editing could be useful also exploited to combine several pathogen recognition sites (PRSs) into a novel engineered R-gene able to mount resistance to some conserved pathogen effectors and/or PAMPs. Indeed, several works have shown that certain motifs are sufficient to determine resistance in the plant host. The highly conserved EDVID motif of the CC domain has been shown to be important for the function of the *R* proteins (Rairdan et al., 2008). Other studies revealed that overexpression of the isolated Toll/interleukin-1 receptor (TIR) domains of several Nibbler proteins is sufficient to trigger a hypersensitive reaction (Zhang et al., 2004; Swiderski et al., 2009; Bernoux et al., 2011; Collier et al., 2011; Maekawa et al., 2011). Furthermore, several studies showed that modular assembly of subdomains from different PRRs is used to form functional receptors. Indeed, the extracellular-leucine-rich repeat (eLRR) receptor kinases of the *EFR* receptor were replaced by corresponding parts from different families or species (for example from *FLS2* or *XA21*), broadening its spectrum against diverse pathogens (Albert et al., 2010; De Lorenzo et al., 2011; Schwessinger et al., 2015). Knowledge gathered from one *R*-gene could be exploited to improve the candidates from other plant species to rapidly deliver agronomically useful resistance genes. A promising approach to improving disease resistance could be achieved by combining engineered *R*-genes in the same cultivar for conferring resistance to different pathogens (Piquerez et al., 2014).

## Additional Genome-Editing Targets for Disease Resistance in Crop Plants

**FIGURE 2:**
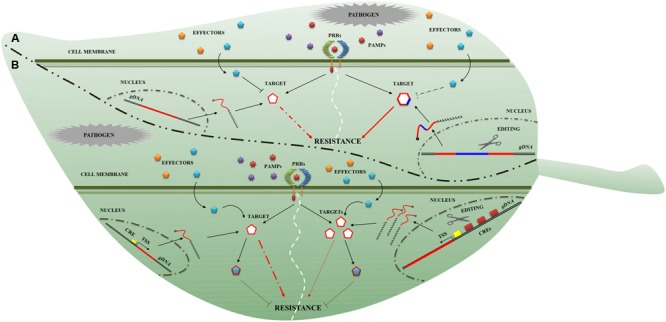
**Novel genome editing targets for plant disease resistance breeding.** Two genome editing applications **(A,B)** to obtain resistance plant are presented. **(A)** In the first example, the modification (blue line) of the effector-target to restore resistance is proposed. The target gene sequence (red line on the left side) is modified (red-blue line on the right side) to impede the effector- (cyan pentagon) target (red-blue hexagon) interaction. **(B)** The second example describes the possibility of modifying the effector-target promoter. In particular, it is depicted a cis-acting regulatory element (CRE; yellow rectangle on the left side) that controls the gene-expression of effector-target (curve red line) and product release (cyan pentagon). On the right, the increase of CREs (red rectangles on the right side) enhances the effector-target expression (curve red lines) and product release (cyan pentagons) conferring a partial resistance (thin red arrows). The dashed red arrows in **(A,B)** panels and dashed black line in **(A)** indicate an interrupted connection between components involved in the resistance process.

One way to achieve broader spectrum resistance is to make use of PRRs. As with the identification of NLRomes, efforts have been made to identify PRRomes (Tang et al., 2010; Andolfo et al., 2013). In addition, detailed knowledge of plant immunity signaling will enable the construction of a novel, resilient immune response network in plants. It is well known that phytopathogens secrete effector proteins that suppress plant immunity (**Figure 2A**). Effector-target genes have great potential in breeding for plant disease resistance (Gawehns et al., 2013). GETs could be used to rewrite the effector-target sequence to avoid their molecular interaction (**Figure 2A**) and to modify the interaction during IAC. Indeed, it was highlighted that a single amino acid change in the effectors (such as in *Phytophthora infestans EPIC1* and in *Phytophthora mirabilis PmEPIC*) and in their corresponding targets (in tomato PLCP and potato RCR3) impairs interaction (Dong et al., 2014). Moreover, the sequence variations in effector targets may cause quantitative variations in resistance phenotypes (Niks et al., 2015). Some important components of immunity, such as *RIN4*, are targets of effectors and might be successfully manipulated by GETs. A *rin4* mutant exhibits increased resistance to the oomycete *Peronospora parasitica* and the bacteria *Pseudomonas syringae* (Luo et al., 2009) in *A. thaliana*. The effector may suppress immunity through attenuation ofHTR, acting on hormone synthesis routes, which are required for resistance to many pathogens. GETs might be used to target the negative regulators of HTR. Indeed, impaired function of negative regulators of the salicylic acid (SA) response (such as the MAP kinase *MPK4*) leads to increased resistance in *A. thaliana* against *Pseudomonas syringa*e and *Peronospora parasitica* (Petersen et al., 2000). Some pathogen effectors could target the host cell physiology through ubiquitination. Since this process contributes crucially to plant immunity, it could be engineered by genome editing. Several studies showed that the knockout of host ubiquitin ligase increased resistance to biotrophic pathogens in *A. thaliana* (Trujillo et al., 2008) and to *Phytophthora infestans* in potato (Bos et al., 2010). The effectors not only interfere with IAC surveillance system but can also modify the plant defense transcriptome more directly. DNA target fragments within the regulatory sequences upstream of the genes that determine resistance to pathogens could be modified as shown in **Figure 2B**. Indeed, early works demonstrated that insertions of short donor sequences can be achieved through the CRISPR/Cas9 system (Li et al., 2013; Shan et al., 2013). It would thus be possible to reprogram the promoter, inserting cis-regulatory elements (CREs) to enhance transcription (**Figure 2B**). Recently, it was demonstrated that the overexpression of a mutant of the phosphatase catalytic subunits (PP1c-1) attenuates infection from *Phytophthora infestans*, interacting with the RXLR effector (Boevink et al., 2016).

In general, GETs allow plant resistance to be modulated by acting on the immunity players, improving the performance of important crops for a sustainable agriculture. Such technologies allow specific mutations to be introduced into effector targets, reducing the pleiotropic effects of complete gene deletion and help to bring about gain-of-function mutations that may promote the use of a quantitative grading of resistance as a valuable approach to protecting crops.

## Author Contributions

GA and PI were primarily involved in drafting the manuscript and producing the figures. LF critically read the manuscript and improved the text. ME conceived the study, drafted and edited the text and coordinated the work. All of the authors read and approved the final manuscript.

## Conflict of Interest Statement

The authors declare that the research was conducted in the absence of any commercial or financial relationships that could be construed as a potential conflict of interest.

The reviewer CM and handling Editor declared their shared affiliation, and the handling Editor states that the process nevertheless met the standards of a fair and objective review.
